# Treatment of MIS-C in Children and Adolescents

**DOI:** 10.1007/s40124-021-00259-4

**Published:** 2022-01-08

**Authors:** Sanaa Mahmoud, Mostafa El-Kalliny, Alyaa Kotby, Mona El-Ganzoury, Eman Fouda, Hanan Ibrahim

**Affiliations:** 1 Division of Allergy and Immunology, Department of Pediatrics, Ain Shams University, Cairo, Egypt; 2 University of Pikeville, Kentucky College of Osteopathic Medicine, Pikeville, KY 41501 USA; 3 Department of Molecular, Cellular, and Developmental Biology, University of Colorado Boulder, Boulder, CO 80309 USA; 4 Medical Scientist Training Program, University of Colorado Anschutz Medical School, Aurora, CO 80045 USA; 5 Division of Cardiology, Department of Pediatrics, Ain Shams University, Cairo, Egypt; 6 Division of Pulmonology, Department of Pediatrics, Ain Shams University, Cairo, Egypt; 7 Division of Critical Care, Department of Pediatrics, Ain Shams University, Cairo, Egypt

**Keywords:** Multisystem inflammatory syndrome in children, MIS-C, Therapy, Intravenous immunoglobulin (IVIG), Corticosteroids, Thromboprophylaxis

## Abstract

**Purpose of Review:**

Different treatment approaches have been described for the management of COVID-19-related multisystem inflammatory syndrome in children (MIS-C), the pathogenesis of which has not yet been fully elucidated. Here, we comprehensively review and summarize the recommendations and management strategies that have been published to date.

**Recent Findings:**

MIS-C patients are treated with different regimens, mostly revolving around the use of immunomodulatory medications, including IVIG and glucocorticoids as first-tier therapy. Refractoriness to IVIG and glucocorticoids warrants a step-up of immunomodulatory therapy to biologic agents such as anakinra, tocilizumab, and infliximab.

**Summary:**

We review the current evidence regarding the use of monotherapy versus combination therapy, as well as the current recommendations for assessing thrombotic risk and administering antiplatelet and anticoagulant therapy. We anticipate that future studies will provide evidence for management plans that maximize short- and long-term outcomes.

**Supplementary Information:**

The online version contains supplementary material available at 10.1007/s40124-021-00259-4.

## Introduction

Evolving evidence and rapidly expanding information are reshaping our understanding of the novel multisystem inflammatory syndrome in children (MIS-C), defined by the CDC and the WHO on the basis of fever, laboratory markers of inflammation, multisystem (≥ 2) organ involvement, and temporal relationship to severe acute respiratory syndrome coronavirus 2 (SARS-COV-2) (WHO, CDC).

Although MIS-C can be severe, it is relatively uncommon, with an estimated incidence of 2 per 100,000 individuals less than 21 years old [1]. To date, in the absence of randomized controlled clinical trials, there remains a paucity of high-quality evidence for management, resulting in variability across clinical practice worldwide. However, guidelines for the management of patients with Kawasaki disease (KD) and similar conditions, in addition to consensus guidelines based on available literature from observational studies, have helped in the development of the clinical approach for the management of MIS-C. Fortunately, a vast majority of reported patients recover quickly in spite of variations in management.

Current practices and published guidelines for the treatment of MIS-C support the use of intravenous immunoglobulin (IVIG) and/or high-dose corticosteroids as a first-line cornerstone of therapy [1, 2, 3•, 4–8]. In addition to antithrombotic therapy and second-line treatment with different immunomodulatory drugs (e.g., tumor necrosis factor inhibitor, interleukin-1 inhibitor, or interleukin-6 inhibitor), other supportive therapeutic agents are concomitantly used [9, 10].

Clinical decision-making and treatment plans should be modified as new evidence emerges, especially in patients with comorbid conditions. Treatment should be individualized, based on both multidisciplinary consensus approach and expert opinion [2, 3•].

## Methods

A literature search through principal medical databases was carried out, including from PubMed/Medline, OVID, Scopus, Google Scholar, briefings/reports from the World Health Organization (WHO), Centers of Disease Control (CDC), and guidance provided by the Royal College of Paediatrics and Child Health (RCPCH). We used a combination of the keywords “MIS-C,” “Multisystem Inflammatory Syndrome,” “Therapy,” “IVIG,” “Immunomodulators,” “Corticosteroids,” and “Thromboprophylaxis” to retrieve articles published from inception to August 31, 2021. We included all articles which provided sound data or recommendations and were relevant to our objectives. Two independent authors screened articles and comprehensively reviewed and extracted appropriate data. This article does not contain any studies with human or animal subjects performed by any of the authors.

### Overview and Initial Management

**Fig. 1:**
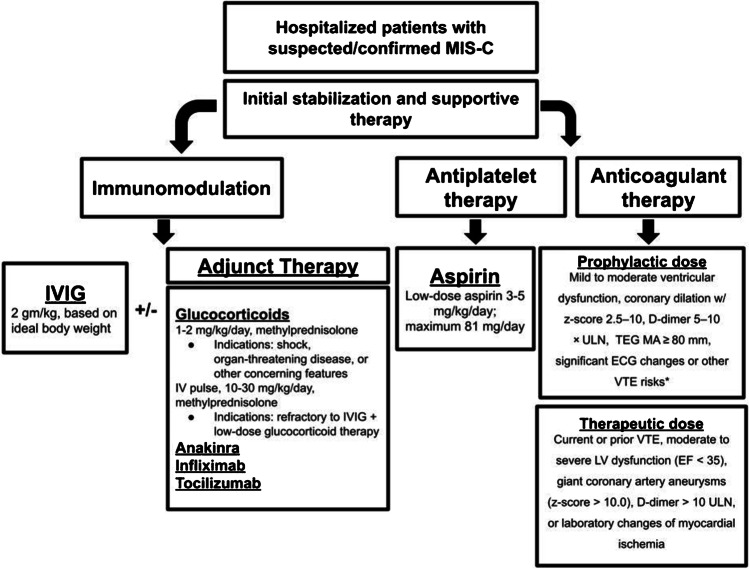
Suggested algorithm for management of MIS-C patients. *VTE risks: obesity, immobilization, age > 12 years, central venous line, asparaginase, malignancy, soft tissue infection, thrombosis in family. Abbreviations: IVIG, intravenous immunoglobulin; ULM, upper limit normal; TEG MA, thromboelastography maximal amplitude; ECG, electrocardiography; VTE, venous thromboembolism; LV, left ventricular; EF, ejection fraction

All children meeting the CDC case definition criteria for MIS-C should be monitored in the hospital with possible admission to the PICU and early involvement of a multidisciplinary team. Children meeting the criteria but not requiring hospitalization (e.g. mildly ill with stable vital signs and unremarkable organ dysfunction) should not be considered as MIS-C but should undergo careful outpatient diagnostic evaluation with close clinical and laboratory follow-up, given the risk of progression to a MIS-C diagnosis, especially as inflammatory markers can take time to rise. Supportive care is based on the severity of symptoms. Those presenting with cardiorespiratory compromise or shock often require careful fluid resuscitation, inotropic support, non-invasive, or invasive mechanical ventilation, in addition to standard PICU care [11]. Some children present with vasodilatory shock, which may be refractory to fluid repletion and require vasopressor support, with epinephrine, norepinephrine, or dopamine. If there are signs of tissue hypoperfusion and cardiac dysfunction despite high doses of catecholamines, inotropes such as dobutamine, levosimendan, or milrinone should be considered, although one study commented on avoiding milrinone due to concern of peripheral vasodilation [12]. Pending blood culture results, empirical use of broad-spectrum antibiotics, as indicated by institutional guidelines for sepsis, is recommended for MIS-C patients who present with signs of shock. Antimicrobial therapy should be refined or de-escalated on the basis of clinical course and microbiological findings. Intravenous immunoglobulin (IVIG), glucocorticoids, and biologic agents constitute the main therapeutic modalities, with different combinations depending on the treatment center (Fig. 1) [13•, 14]. The ultimate aims of management are to decrease systemic inflammation and restore organ function, with the goal of preventing long-term sequelae such as persistent cardiac dysfunction [15].

### Immunomodulatory Management

Although the exact pathogenesis of MIS-C remains elusive, virus-induced post-infective immune dysregulation appears to play a predominant role, with MIS-C commonly developing 2–6 weeks after infection [16]. Proposed immunologic mechanisms include but are not limited to superantigen-like activation of the immune system, and autoantibody production resulting in activation of Fcy receptors on neutrophils and macrophages, causing secretion of pro-inflammatory cytokines [17–19]. Accordingly, immunomodulation is a cornerstone of treatment, with consensus guidelines recommending a stepwise progression of immunomodulatory therapies starting with the first-tier therapy, IVIG, which can sometimes be used as needed before full diagnostic evaluation is completed. Glucocorticoids are used in low doses as adjunctive therapy in patients with the moderate-to-severe disease or in high doses as intensification therapy in patients with refractory disease. Biologics such as anakinra, tocilizumab, or infliximab have been used as adjunct therapy with success by multiple centers; however, based on the latest recommendations of the American College of Rheumatology (ACR) and others, they are used in cases refractory to first-line treatments [3•, 20].

### Intravenous Immunoglobulin (IVIG)

The adoption of IVIG as a treatment in MIS-C was based on the similarities that MIS-C shares with Kawasaki disease, including coronary artery aneurysms (reported in 9–24% of MIS-C patients) and findings of myocarditis in many patients [9, 21, 22]. The mechanism of action of IVIG includes inhibition of complement deposition, enhancement of regulatory T cells, and accelerated clearance of autoantibodies [23]. Although there have been no randomized studies of the efficacy of IVIG, reviews and observational studies have reported efficacious outcomes with an increasing frequency of IVIG use over time [24, 25]. Multiple later studies have reported a frequency of IVIG use greater than 90%, with as little as 0% mortality [26, 27].

Consensus guidelines support the use of high-dose IVIG for all hospitalized patients, administered in a single dose at 2 gm/kg based on ideal body weight (max 100 gm) [3•, 28•]. Cardiac function and fluid status should be assessed before IVIG administration; patients with depressed cardiac function or vasodilatory/distributive shock should be closely monitored and the use of diuretics should be considered [3•, 25]. In some patients with ventricular dysfunction, IVIG dose can be given through slower infusion or divided into 1gm/kg daily over 2 days, to avoid fluid overload or further cardiac decompensation [3•, 25]. A second dose of IVIG is considered in some recommendations [28•], although, in version 2 of the ACR recommendations, a second dose of IVIG is not recommended for all patients, given the risk of volume overload, hemolytic anemia, aseptic meningitis, neutropenia, and other side effects [3•].

### Corticosteroids

Corticosteroids are one of the few validated therapeutics in critically ill adult patients with COVID-19 [29, 30]. The rationale for using steroids are their anti-inflammatory properties and frequent use in Kawasaki disease and other inflammatory disorders [31]. A variety of observational studies suggest that corticosteroids are beneficial in MIS-C patients, possibly due to their effect in treating features of cytokine storm and the frequent finding of a shock-like presentation [14, 22].

Two recent studies examined the effectiveness of IVIG monotherapy in comparison to combination therapy with IVIG and glucocorticoids in adult COVID-19. In a study by The Overcoming Covid Consortium, which analyzed data from a cohort of 596 patients, combination therapy was associated with a lower risk of cardiovascular dysfunction and less use of adjunctive immunomodulatory treatments later on, in contrast to IVIG monotherapy [22]. These findings are in line with previous studies suggestive of similarly improved outcomes with combination therapy [14, 32•]. In contrast, a study from the international Best Available Treatment Study (BATS) consortium analyzed data from a cohort of 651 patients and compared three treatment groups: IVIG alone, a combination of IVIG and glucocorticoids, or glucocorticoids alone. There were no statistically significant differences in primary outcomes of mechanical ventilation, inotropic support, death, or reduction in disease severity [33]. However, in patients who received IVIG plus glucocorticoids, there was a significantly decreased risk of escalation of immunomodulatory treatment, compared to patients who received IVIG alone. The length of hospital stay and time until improvement in inflammatory markers was similar across all three treatments. Importantly, neither of these studies definitively answer the question about the most effective single or combination treatment, and neither of these studies addressed the treatment of MIS-C [34].

The American College of Rheumatology (ACR) consensus guidelines for MIS-C suggest that intravenous low-to-moderate dose corticosteroids (methylprednisolone 1–2 mg/kg/day, typically in two doses) should be used alongside IVIG in patients demonstrating shock or organ-threatening disease (Fig. 1) [3•]. Methylprednisolone is most commonly used and demonstrated better results than dexamethasone in treating hospitalized hypoxic adult COVID-19 patients [35], but other steroids that can be used are dexamethasone (0.15–0.4 mg/kg/day, p.o. or IV) and prednisone (1–2 mg/kg/day, p.o.) [22, 36]. This low-to-moderate dose of glucocorticoids may also be considered for administration alongside IVIG in patients who have not yet developed shock or severe end-organ involvement but present with concerning features such as ill appearance, highly elevated BNP levels, or unexplained tachycardia [3•, 28•]. In patients refractory to initial treatment with IVIG and low-to-moderate dose corticosteroids, corticosteroid treatment can be intensified to pulse doses of methylprednisolone (10–30 mg/kg/day). Refractory disease is defined as persistent fever and/or ongoing significant end-organ involvement.

Children with MIS-C may require a prolonged course of treatment for several weeks to avoid rebound inflammation. Glucocorticoid-related complications are predominantly hypertension and hyperglycemia, with one study reporting an incidence of 4% in patients who received glucocorticoids in any combination [33]. Upon resolution of fever and clinical improvement, treatment can be transitioned to an equivalent oral dose of prednisolone or prednisone [3•]. Gastrointestinal prophylaxis should be administered during corticosteroid treatment according to institutional guidelines [13•]. All patients should be slowly weaned from treatment depending on the clinical course and the results of serial laboratory testing and cardiac assessment [3•, 28•].

### Anti-cytokine Therapy

Anti-cytokine agents are often successfully used as an initial adjunct therapy to IVIG. However, data evaluating the efficacy of using targeted biologic agents is not yet available [34, 37], and later recommendations emphasize the use of biologics primarily for cases refractory to treatment with IVIG and corticosteroids [3•, 20, 28•].

Immunomodulatory treatment with biologic agents includes interleukin-1 (IL-1) blockade with anakinra, interleukin-6 (IL-6) blockade with tocilizumab, and/or monoclonal antibodies to tumor necrosis factor (TNF) with infliximab. There have been no studies supporting routine serial cytokine level assessment in the guidance of cytokine-targeted therapy (CTT) [28•].

### Anakinra

IL-1 is an important effector cytokine of innate immunity that is produced by alveolar type II cells upon infection with SARS-CoV [38, 39]. Anakinra is a recombinant human IL-1 receptor antagonist (IL-1ra) used for rheumatoid arthritis and other inflammatory conditions, including neonatal-onset multisystem inflammatory disease (NOMID) [40]. Studies of anakinra in adult COVID-19 patients have demonstrated improvements in respiratory function and inflammatory markers as well as mortality and the need for invasive mechanical ventilation [41–45].

Anakinra should primarily be considered in MIS-C patients with disease refractory to IVIG and/or corticosteroids, or in patients with contraindications to steroids, especially in cases of MIS-C with features of macrophage activation syndrome [3•]. However, initiation of anakinra as an adjunct therapy to IVIG or before invasive mechanical ventilation may be beneficial [3•]. Schlapbach and colleagues suggest starting dosing at 2–3 mg/kg q12 h s.c. (total of 4–6 mg/kg/day, max. 100 mg/dose) [28•]. Liver function in patients who receive anakinra should be monitored, due to the risk of increased serum transaminases and hepatitis [46]. However, its short half-life (4–6 h) and quick onset of action allow for frequent reassessment and rapid discontinuation if adverse reactions are observed [28•, 47]. Other side effects include neutropenia, leukopenia, thrombocytopenia, headache, abdominal pain, nausea/vomiting, and diarrhea.

In the case of no clinical or biochemical improvement to anakinra treatment within 24–48 h, other targeted immunomodulators such as tocilizumab or infliximab can be considered [28•]. In the case of clinical improvement, anakinra can be discontinued after 48–72 h with or without tapering, according to institutional practice.

### Tocilizumab

Tocilizumab is a humanized anti-human IL-6 receptor antibody that is used in juvenile idiopathic arthritis and other inflammatory conditions. Initially, tocilizumab appeared to be an effective option for treating adult COVID-19 [48, 49]; however, randomized, double-blind, placebo-controlled trials showed no clear benefit of tocilizumab in preventing intubation or death in hospitalized adult patients with COVID-19 [50, 51]. In addition, safety concerns include an increased risk for infections, and a long half-life (150 h) [28•].

For these reasons, tocilizumab is not recommended for the majority of MIS-C patients; however, it can be considered for use in children with the life-threatening disease in whom prior therapy, including anakinra, has not been effective [3•, 28•]. Given the long half-life, it is usually administered as a single IV dose (< 30 kg: 12 mg/kg IV; ≥ 30 kg: 8 mg/kg IV; max.: 800 mg).

### Infliximab

Levels of tumor necrosis factor-alpha (TNFα), a pro-inflammatory cytokine, have been demonstrated to be associated with severe KD complications and to differ between patients with MIS-C and patients with severe COVID-19 [9, 52]. Some studies utilizing infliximab, the monoclonal anti-TNFα antibody, have reported efficacy in the treatment of MIS-C [53]. However, currently, infliximab is not recommended for use in a majority of patients and can be considered on a case-by-case basis in patients who have failed to respond to prior treatments or in patients who present with comorbid conditions such as Crohn’s disease [3•, 54].

### Thromboprophylaxis and Thrombosis

#### Thromboembolism in MIS-C

Treatment strategy for thromboprophylaxis in MIS-C patients presents multiple challenges since pediatric VTE pathophysiology and risk factor definitions differ from those of adults [55]. MIS-C coagulopathy, in particular, is thought to arise from marked host inflammatory response, cytokine-mediated endotheliopathy, and platelet activation [56, 57•]. Multicenter studies have shown an incidence of MIS-C associated thromboembolic events (e.g., pulmonary embolism (PE), deep venous thrombosis (DVT), thrombotic microangiopathy (TMA), and arterial thrombosis including stroke) ranging from 6.5 to 8%, with one multicenter study showing a mortality rate of 28% in those who developed thrombosis [7, 58–60]. However, the reported incidence likely underestimates the true baseline risk of thrombosis, considering that many patients in these studies received thromboprophylaxis during hospitalization [60]. The recent approaches of Sharathkumar et al. and Bansal et al. together provide comprehensive practical guidance for addressing thrombosis and thromboprophylaxis risk assessment as well as management in most MIS-C cases [57•, 61•].

#### Thromboembolism Risk

The first step in deciding on the proper use of antithrombotic therapy resides in the assessment of condition severity, VTE risk factors, location of the patient (outpatient, regular ward, or PICU), and oxygen requirement [57•, 60, 62, 63]. Detailed risk factors of VTE in hospitalized children with COVID-19 are outlined by Goldenberg and colleagues [64]. All management plans consider evaluating all or most of the characteristic coagulation profile, including D-Dimer (usually assessed every 24–72 h), PT/INR, PTT, fibrinogen, platelet count, lactate dehydrogenase (LDH), thromboelastography with platelet mapping (TEG with PM), or rotational thromboelastometry (ROTEM), specifically evaluating for elevated clot strength or maximal amplitude (MA) suggestive of hypercoagulability (MA > 80 mm) [61•]. A D-dimer level above five times the upper limit of normal (ULN) has been suggested as an independent predictive factor that should be incorporated into risk assessment [57•, 61•, 63, 64].

#### Antiplatelet Therapy

Aspirin is used as a thromboprophylaxis in MIS-C due to its role in addressing platelet activation, endothelial damage, and altered flow dynamics [57•, 65]. The use of aspirin was patterned after its use in Kawasaki disease, which appears to share fundamental characteristics with MIS-C, including altered flow dynamics in coronary vessels [33, 65–67].

Low-dose aspirin (3–5 mg/kg/day, maximum 81 mg/day) should be considered in all hospitalized MIS-C patients, unless contraindicated (platelet count < 100,000/mm^3^, fibrinogen < 100 mg/dL, active bleeding, or concern for high risk of bleeding) [3•, 57•, 61•]. Aspirin should be continued for at least 1 month from diagnosis regardless of inflammatory markers and coagulation profile or longer until levels normalize and coronary arteries are demonstrated to be normal [5, 66, 67].

#### Anticoagulant Thromboprophylaxis

In addition to aspirin, the concomitant use of anticoagulation (e.g., low molecular weight heparin, unfractionated heparin (UFH), direct thrombin inhibitor, direct oral anticoagulant) in the absence of other bleeding risks (as exemplified in cases of significant risk of bleeding or platelet count < 20.000/mm^3^), is not contraindicated [57•, 64]. In noncritically ill adult patients hospitalized with COVID-19, therapeutic-dose anticoagulation was superior to usual-care thromboprophylaxis in increasing the probability of survival to discharge with reduced use of cardiovascular or respiratory organ support [68].

For MIS-C, recommendations for low-dose (prophylactic) anticoagulation include mild to moderate ventricular dysfunction, coronary dilation/aneurysm with z-score 2.5–10, D-dimer 5–10 × ULN, TEG MA ≥ 80 mm, or any new significant rhythm abnormalities such as heart block, premature atrial and ventricular contractions, conduction abnormalities, and ST-segment changes [61•]. The 2017 AHA recommendations for the management of Kawasaki disease can be used as guidelines for dosing regimens [65].

Consensus guidelines support the use of LMWH as the anticoagulant of choice for prophylactic dosing, which is commonly administered subcutaneously twice daily [69–71]. There is no consensus with regards to monitoring anti-Xa for prophylactic dosing, but one observational study suggests improved efficacy and safety with dosing titrated according to anti-Xa levels [63]. Unfractionated heparin, which has both hepatic and renal clearance and can be reversed with protamine sulfate, is an alternative if LMWH is contraindicated in cases of severe renal impairment. Heparin-induced thrombocytopenia has been reported in COVID-19 patients and should be considered if a drop in platelet count is observed [57•, 65, 72].

Although not yet FDA approved for children, direct oral anticoagulants (DOACs) are increasingly used in patients with normal renal function and have no contraindications to their use. They can be considered for use as prophylactic anticoagulation [57•, 61•].

Indications for initiating high-dose (treatment) anticoagulation include current or prior VTE, moderate-to-severe LV dysfunction (EF < 35), giant coronary artery aneurysms (z-score > 10.0), D-dimer > 10 ULN, and laboratory changes consistent with myocardial ischemia [3•, 57•, 61•]. Patients can be switched to low-dose anticoagulation as echocardiogram and laboratory values improve.

For hospitalized patients with less severe MIS-C who do not have clear indications for anticoagulation, the approach to management should be individualized and tailored according to the patient’s risk for thrombosis and risk of bleeding.

#### Post-discharge Anticoagulation

Consensus-based guidelines support the use of prophylactic-dose anticoagulation for around 30 days post-discharge, with consideration of shorter or longer duration based on resolution or progression of clinical risk factors and laboratory values [3•, 57•, 61•, 65].

Patients with MIS-C and documented thrombosis or EF > 35% should receive therapeutic anticoagulation until at least 2 weeks after discharge from the hospital [3•]. Indications for longer outpatient therapeutic dosing include the following: CAAs with a z-score of > 10.0 (indefinite treatment), documented thrombosis (treatment for ≥ 3 months pending thrombus resolution), or ongoing moderate-to-severe LV dysfunction [3•]. For children with persistent risk factors, such as continued immobility, presence of central line, and significant D-dimer elevation, treatment should be continued until risk factors are no longer present [3•, 57•].

#### Other Therapies

The use of extracorporeal membrane oxygenation (ECMO) varies considerably and should be considered in cases of ARDS or cardiorespiratory failure refractory to conventional management [57•, 73].

Therapeutic plasma exchange (TPE) is a well-known therapy designed to remove high molecular weight substances from the blood, with many non-specific mechanisms, including the removal of cytokines and autoantibodies. TPE is not routinely used in MIS-C, but has been used as a rescue immunomodulatory treatment in critically ill patients who have not responded to third-line immunomodulatory treatments or in cases in which biologic drugs are not available [74–77].

Remdesivir has not been well studied in the management of MIS-C and should not routinely be used; however, it can be considered for compassionate use on a case-by-case basis, particularly for those who are PCR positive [11, 57•].

### Limitations

Limitations of current recommendations include reliance on observational studies and less applicability to low-resource settings in which access to complex diagnostic tools, IVIG, biologicals, and other therapies are more limited. The pharmacological approach that has been described here is also generally limited to those hospitalized with moderate-to-severe disease. Long-term outcomes of MIS-C patients remain unknown.

## Conclusions

Here, we have reviewed the available literature on MIS-C therapies and presented treatment options, including the use of immunomodulators, thromboprophylactic agents, and other therapies. The timely and swift administration of immunomodulatory therapy improves outcomes and decreases the need for escalation of life-supportive care. In our review of data over time, we observed that patients treated according to later developed recommendations underpinned by prompt and aggressive immunomodulation, appeared to demonstrate lower mortality and lesser need for supportive care such as ECMO, compared to initial cohorts of patients who were treated with less aggressive immunomodulation.

Nevertheless, there remain significant knowledge deficits regarding the optimal selection of immunomodulatory therapies, especially with regards to long-term cardiac sequelae. A refined classification of the spectrum of different phenotypes and subtypes of illness related to MIS-C will likely allow for more precise determination of optimal therapies for specific, defined subgroups of patients.

Finally, the information and recommendations summarized in this article should not supersede the role of independent clinical judgment. Each patient will need an individualized, multidisciplinary approach in the context of his/her clinical course.

## Supplementary Information

Below is the link to the electronic supplementary material.

Supplementary file1 (JPG 39 KB)
