# Supplementary figures and images for: Treatment of MIS-C in Children and Adolescents
Source: Curr Pediatr Rep. 2022 Jan 8;10(1):1–10. doi: 10.1007/s40124-021-00259-4 (PMC8741532; doi:10.1007/s40124-021-00259-4)

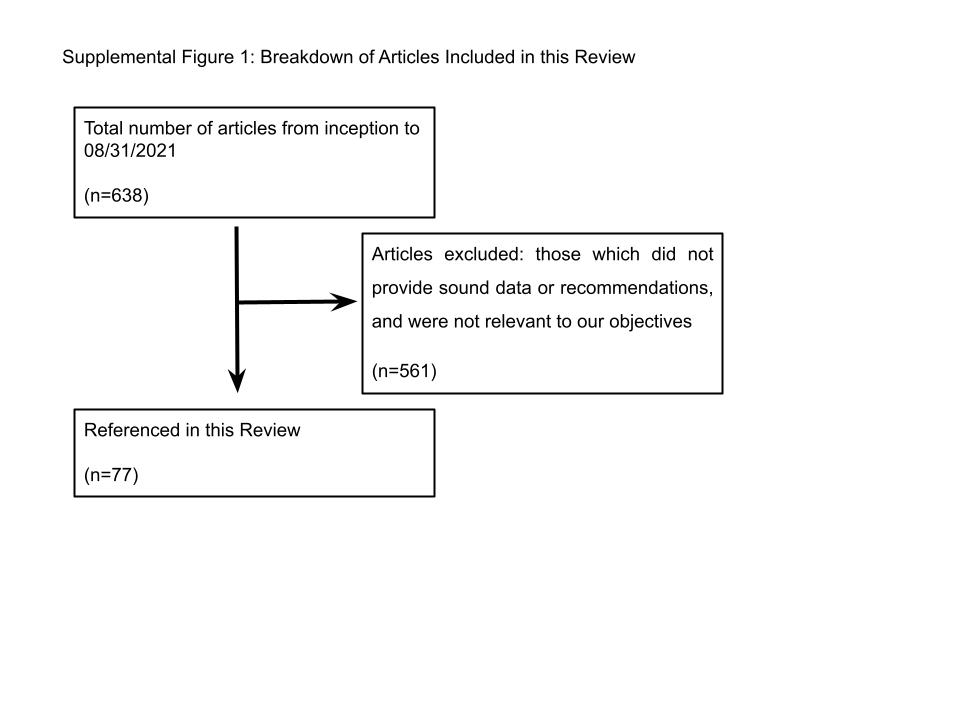

Supplement: Supplementary file 1 — Supplementary file1 (JPG 39 KB) [file 40124_2021_259_MOESM1_ESM.jpg]
